# *Spaghetti Tracer*: A Framework for Tracing Semiregular Filamentous Densities in 3D Tomograms

**DOI:** 10.3390/biom12081022

**Published:** 2022-07-23

**Authors:** Salim Sazzed, Peter Scheible, Jing He, Willy Wriggers

**Affiliations:** 1 Department of Computer Science, Old Dominion University, Norfolk, VA 23529, USA; ssazz001@odu.edu (S.S.); psche004@odu.edu (P.S.); 2 Department of Mechanical and Aerospace Engineering, Old Dominion University, Norfolk, VA 23529, USA

**Keywords:** actin, cryo-electron tomography, segmentation, dynamic programming, denoising, missing wedge, tomogram simulation

## Abstract

Within cells, cytoskeletal filaments are often arranged into loosely aligned bundles. These fibrous bundles are dense enough to exhibit a certain regularity and mean direction, however, their packing is not sufficient to impose a symmetry between—or specific shape on—individual filaments. This intermediate regularity is computationally difficult to handle because individual filaments have a certain directional freedom, however, the filament densities are not well segmented from each other (especially in the presence of noise, such as in cryo-electron tomography). In this paper, we develop a dynamic programming-based framework, *Spaghetti Tracer*, to characterizing the structural arrangement of filaments in the challenging 3D maps of subcellular components. Assuming that the tomogram can be rotated such that the filaments are oriented in a mean direction, the proposed framework first identifies local seed points for candidate filament segments, which are then grown from the seeds using a dynamic programming algorithm. We validate various algorithmic variations of our framework on simulated tomograms that closely mimic the noise and appearance of experimental maps. As we know the ground truth in the simulated tomograms, the statistical analysis consisting of precision, recall, and *F*1 scores allows us to optimize the performance of this new approach. We find that a bipyramidal accumulation scheme for path density is superior to straight-line accumulation. In addition, the multiplication of forward and backward path densities provides for an efficient filter that lifts the filament density above the noise level. Resulting from our tests is a robust method that can be expected to perform well (*F*1 scores 0.86–0.95) under experimental noise conditions.

## 1. Introduction

In cryo-electron tomography (cryo-ET), frozen-hydrated cell samples are mounted on a rotating specimen holder to capture multiple views of biological cells in their native environment. After 3D reconstruction, the resulting tomograms provide 3–5 nm resolution views of the cell’s ultrastructure, down to the level of macromolecular assemblies and individual molecules. However, even after image processing, the tomograms exhibit considerable noise because the electron dose is limited to prevent radiation damage.

There are also blurring artifacts visible in the 3D reconstruction because of the limited tilt range of the specimen holder that masks out a wedge of the transformed image data in Fourier space [1]. In recent years, several computational groups have focused on automatically detecting and segmenting biomolecular shapes in such tomograms by using techniques such as deep learning [2,3,4]. Furthermore, a number of toolboxes have been developed for tomography analysis [5,6,7]. These efforts have mainly been aimed at improving the signal-to-noise ratio by averaging multiple particles in subtomogram averaging [8]; however, the averaging approach is unsuitable for long cytoskeletal filaments that exhibit variable shapes.

Our own work over the past decade has mainly focused on developing specialized techniques for the automated detection and modeling of cytoskeletal filaments such as actin [1,9,10]. The orientation of actin filaments within cells is of considerable biological importance because the filaments organize the structure of functional cell appliances such as focal adhesions, bacterial comet tails, hair cell stereocilia, lamellipodia, and filopodia. Actin filament detection and analysis has become the focus of optical microscopy in 2D [11,12] and cryo-ET in 3D [13,14,15,16].

Given the noise, missing-wedge artifacts, and large volume of cryo-ET reconstructions, computational tracing methods often require manual intervention [17]. Furthermore, a manual tracing of cytoskeletal filaments is labor intensive [18]. To achieve a more reproducible and efficient analysis, we have developed several fully automated approaches over the past decade that do not require manual intervention. These approaches were motivated by the biological application requirements of our experimental collaborators.

For example, in *Dictyostelium discoideum* filopodia, the packing density is relatively low, meaning that individual actin filaments are well separated and show random orientation. We developed the *voltrac* tool to find the seed locations of filaments using a genetic algorithm-based search of a population of cylindrical templates [9]. The filaments can then be traced, starting from the seeds, by using a bidirectional search that can follow curved paths, as long as the filaments are separated. In contrast, the shaft region of hair cell stereocilia is comprised of densely packed bundles that can be traced with *bundletrac* [10], starting from user-provided seed points, here by taking advantage of the hexagonal packing symmetry orthogonal to the filament axis.

In this work, we analyze the taper region of hair cell stereocilia [18]. Actin bundles in this region exhibit an intermediate density that imposes a certain regularity and mean direction; however, individual filaments can deviate from this mean direction. Moreover, because of the proximity of the filaments, the missing-wedge effect fuses adjacent densities, seemingly impeding their direct tracing. This problem led us to earlier developing a template-based deconvolution [1] to correct the missing-wedge artifacts prior to the tracing; however, this approach required expensive numerical techniques (up to a week of run time), and it came at the price of interpreting the entire tomogram (including membranes, other biomolecules, and noise) as filaments, leading to false-positive predictions.

In a recent workshop paper [19], we showed that it is possible to achieve a significantly faster tracing of dense, semiregular filaments (as in the challenging intermediate density case of the stereocilia taper region [18]) by combining denoising with tracing in a single dynamic programming (DP)–based framework. This approach was particularly promising because of the small number of false-positive predictions. However, earlier work has called for additional tests and optimizations, which we provide in the present paper. For example, the DP approach relied on a novel design of a bipyramidal path density (*PD*) accumulation scheme, and thus we also tested a more conventional straight-line *PD* accumulation to demonstrate the need for our innovation.

**Figure 1:**
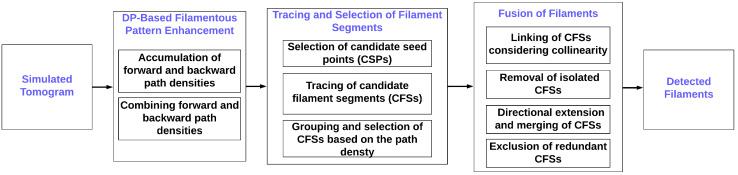
The filament-tracing framework originally developed in [19]. The enhancement step filters the tomogram by utilizing a cumulative path-based density-contrast enhancement algorithm. This relatively slow preprocessing step raises the intensity values of filaments to make them stand out better from the noise. Subsequently, the filaments can be quickly generated in a bottom-up manner from candidate seed points (CSPs), which is followed by tracing short candidate filament segments (CFSs) from the CSPs. The large number of short CFSs are then refined and fused to generate the detected output filaments.

For the noisy density maps arising in cryo-ET, we recommended in [19] a denoising filter as a preprocessing step; the filter was based on the same DP algorithm that we developed for the filament tracing (Figure 1). Although computationally more expensive than the later tracing, the preprocessing can strengthen the underlying filamentous structures against the noise for better visualization and automatic extraction [19]. Enhancement techniques have also been proposed by other groups as well [20,21]. To provide further justification for its inclusion in the workflow (Figure 1), we investigated the filament-tracing performance at various noise levels in the presence and absence of this preprocessing step.

The denoising filter relies on the most efficient blending of the forward and backward accumulated PDs. We empirically found that the filament contrast can be enhanced when requiring both PDs to be simultaneously of a high value. The simplest way to implement this in [19] was by multiplying the PDs (essentially, using the square of the geometric mean). A squared density, however, has no biological meaning. Furthermore, it was not clear if the product offered the most discriminating performance against the noise. Therefore, we have explored three additional blending functions in this paper that do not yield a squared density: the arithmetic mean, geometric mean, and minimum.

We introduced a new automatic method for seed point generation based on a spatial decomposition of the volume that specifically designed for our semiregular filament bundles [19]. These seeds provide the starting points for the traces grown from the seeds. The nascent filaments are processed in various groupings, screenings, and fusion stages (Figure 1), eventually yielding the final interpretation.

The filament predictions can then be evaluated in terms of true and false positives or negatives and statistical criteria, such as recall, precision, and *F*1 score. The statistical validation requires a known ground truth; therefore, we used only simulated tomograms modeled to closely match the noise level, noise color, and missing-wedge effects of an experimental tomogram. The results provide a quantitative justification and optimization of the various algorithmic modifications we have explored.

## 2. Methods

This section first introduces how our simulated tomograms were built from an existing manual tracing and how the map noise levels for our validation tests were calibrated against the existing experimental map. The simulated tomograms were then subjected to the main filament-tracing framework, which was developed in [19] and is depicted in Figure 1. The framework consists of several denoising, seed generation, tracing, refinement, and fusion steps, which will be described in the following.

### 2.1. Noise Calibration and Simulation of Tomograms

**Figure 2:**
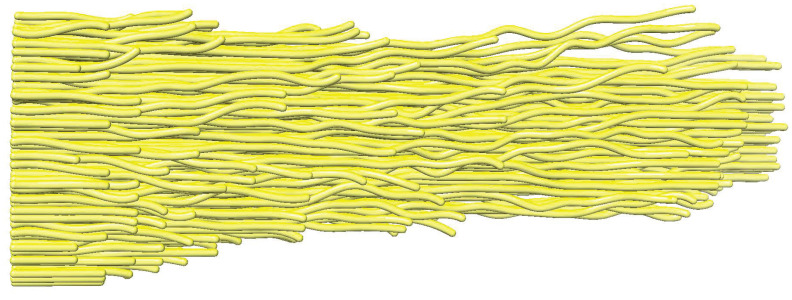
Manually traced filaments in the stereocilia taper region. The manual “spaghetti” model is consistent with the experimentally observed density of the actin filaments [18] in cryo-ET. The true position of actin filaments in the experimental map is not known with complete certainty, but manual tracing can serve as a ground truth for testing our algorithms when using simulated tomograms modeled after the shown filament traces. All molecular graphics figures in the present paper have been prepared with UCSF Chimera [23] and oriented with the +Y direction to the right.

We recently developed a software tool, *TomoSim*, for the realistic simulation of tomograms [22]. The simulation will allow us to test the filament-tracing performance based on a known filament model (Figure 2) and its corresponding experimental tomogram. The initial model can be a manually obtained interpretation of an experimental density map (Figure 2) or automated tracing generated by a computational tracing approach described in the next section. As described in more detail in [22], the simulation aims to faithfully recreate the noise and missing-wedge Fourier space artifacts typically found in cryo-ET. The simulations created for the current paper do not include nonfilamentous biological features, such as membranes.

We started the simulation approach by interpolating the existing model filaments and rasterizing them onto the cubic grid of an experimental map corresponding to the start model. The grid indices i,j, and *k* correspond to the X,Y, and *Z* axes, respectively. We also retained the size and dimensions of the experimental reference map grid in our simulations.

The projected filament traces were then enlarged by convolving the voxel densities D(i,j,k) with a Gaussian-shaped kernel whose dimensions (full width at half maximum 5 nm with a voxel spacing of 0.947 nm, ∼2.01 voxels) were matched to the width of an actin filament. Color-filtered noise was added from a noise map that matched the radial power spectral density (noise color) and the signal-to-noise ratio of an experimental reference tomogram [22]. In a second step, we also matched the noise level in the experimental map by visual appearance by applying an amplification factor of 1.85 to the filament voxels prior to volumization. This additional manual amplification can be seen as subjective, but it helped provide a better visual match to the experimental data than our present automated noise matching, as described in [22]. The manual adjustment accounted mainly for discrepancies between the model and experimental map. For example, the manual tracing might not perfectly match the experimental tomogram positions (which we called the “alignment error” in [22]) or the tomogram might exhibit an inhomogeneous density distribution across the volume due to gaps or helical twist in the actin [18] (whereas our simulated filament densities were perfectly homogeneous along the filament length). In such situations, the automatically calibrated filament signal strength can be weaker than a visual inspection suggests. After noise was added, a wedge was masked out in Fourier space to emulate the missing views from the limited tilt range of the microscope specimen holder.

**Figure 3:**
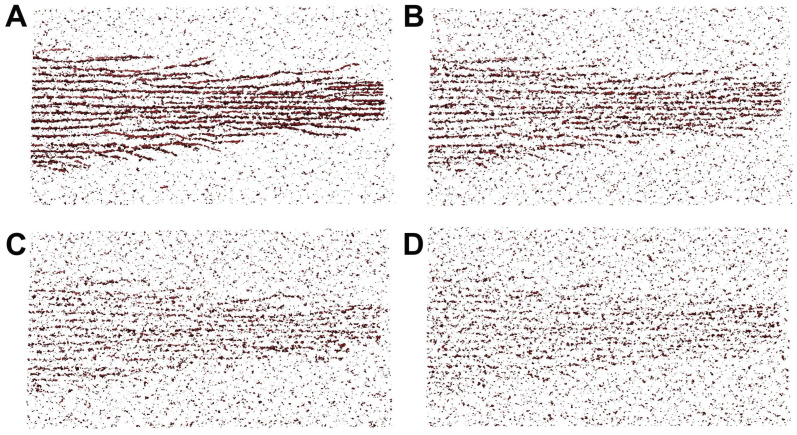
Illustration of the simulated density maps at various noise levels relative to noise in the experimental map [18] (see text). (**A**) noise level 0.40; (**B**) noise level 0.60; (**C**) noise level 0.80; (**D**) noise level 1.00. For the illustration, we used a 10-voxel-thick slab (corresponding to *Z*-indices 120–129, using experimental map voxel spacing 0.947 nm [18]), with an isocontour density threshold of mean plus two times the standard deviation.

To validate the performance of our proposed tracing framework, we have considered simulated tomograms with varying degrees of noise (Figure 3). In our simulations, we used four noise levels ranging from 0.4 to 1.0 (Figure 3). The manually amplified noise level (factor 1.85) was used as an upper bound (worst case experimental noise), which was normalized as a noise level of 1.0 in the current paper. Lower levels were used as an additional scale factor. For example, the 0.6 noise level would be the closest to the automatically matched experimental noise. The lowest 0.4 level might help identify improvements that could be afforded by better quality data in future work.

In summary, the main advantages of using simulated maps were as follows: (i) to provide us with a needed ground truth for validation and (ii) to free us from the above subjective amplification uncertainty (the manual matching is only used once to set the reference level 1.0). Consequently, we can consider a reasonable range of noise levels that we would expect to encounter in experimental maps. The noise level (global scale factor) did not affect the noise color calibration or missing-wedge modeling.

### 2.2. Density Map Preprocessing: Accumulation of Forward and Backward Path Densities

**Figure 4:**
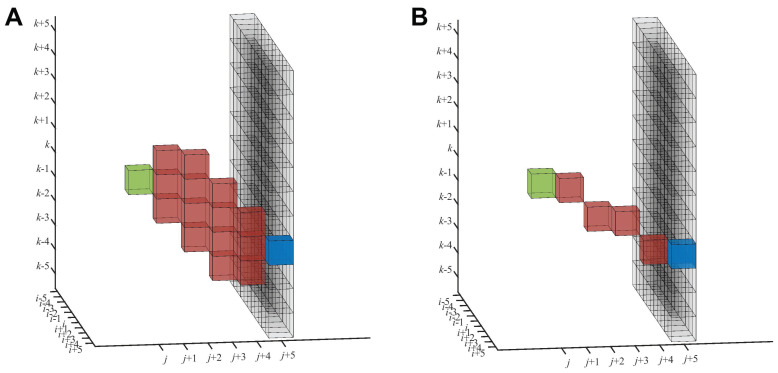
Two path density accumulation schemes were employed in the filament pattern enhancement and CFS tracing steps (Figure 1). (**A**) illustrates how the forward path density (FPD) of length l=5 voxels accumulated from the voxel (i,j,k) (green) using the original DP approach [19] in Equation (1). The accumulation zone (red) is shown for a random target voxel ($i^{\prime },j+l,k^{\prime }$) (blue), but the final FPD has been taken as the maximum among all potential targets (black) at the base of the search pyramid ($i-l\leq i^{\prime }\leq i+l,k-l\leq k^{\prime }\leq k+l$, Equation (1)). (**B**) shows how the FPD accumulated with the alternative straight-line approach (also l=5 voxels) in the CFS tracing (Figure 1).

For cryo-ET maps, we recommended in [19] a denoising filter as a preprocessing step. Our approach assumes that filaments have a mean direction, which, in the current work, is in the *Y* direction, the same as the experimental map [18]. Individual filaments may deviate from the mean direction, so we allowed for an up to a 45${}^{\circ }$ deviation from the dominant axis. For each voxel (i,j,k), here represented in green in Figure 4, the preprocessing step assigns the path density values accumulated following a search window, starting from (i,j,k). This search window originating from (i,j,k) has a pyramidal shape in both directions constrained by the 45${}^{\circ }$ limiting angle. The end points of the search window in the forward direction (fully shown in Figure 3 of [19]) are represented in Figure 4 by the black voxels (the base of the pyramid).

The forward path density FPD(i,j,k;l) (Figure 4A) and backward path density BPD(i,j,k;l) (similar, not shown) are accumulated along certain paths (red) of a fixed length *l*. In this paper, the term “length” is used for the extent in the *Y* direction (mean direction), not the Euclidean length. The length *l* can be tuned to the nominal resolution of the tomogram or separation of the desired features, or it can be used to control the straightness of the filaments. In this work, we set the length of *l* to five voxels, here considering the trade-off between the noise present in the tomogram and shape of the filaments. The originally proposed accumulation scheme (fully shown in Figure 4 of [19]) adds up intensity values within a zone of influence that forms a reverse pyramid with its tip at the target voxel, ($i^{\prime },j+l,k^{\prime }$) (blue in Figure 4). The FPD(i,j,k;l) is then defined as the maximum PD among all the target voxels in the base of the pyramid.

Therefore, the voxels contributing to the FPD(i,j,k;l) form the intersection of two pyramids, the forward-facing search pyramid (Figure 4A), and the reverse influence pyramid with its tip at the blue target voxel (Figure 4 of [19]). This intersection is the accumulation zone, as shown in red in Figure 4A. The accumulation zone is confined to a relatively localized volume reaching from the green origin (i,j,k) to the potential blue target ($i^{\prime },j+l,k^{\prime }$). This localized zone inspired us to also test a simpler approach, where the path densities are accumulated directly along a thin straight line of voxels, as shown in Figure 4B (see below).

For mathematical completeness, we again provide the details of the DP implementation (described above and shown in Figure 4A). Readers familiar with the workshop proceedings [19] may skip the following equations.

#### 2.2.1. Pyramidal Search Window and Maximum Path Density Selection

The voxel densities D(i,j,k) (normalized between 0 and 1) are accumulated as follows toward the base of the forward pyramid (j+l; black in Figure 4) and, similarly, in the backward direction (j−l; not shown), from which the maximum values can be selected:

(1) $$\begin{array}{cc} \; & FPD\left(i,j,k;l\right)=\max_{\begin{array}{c} i-l\leq i^{\prime }\leq i+l\\ k-l\leq k^{\prime }\leq k+l \end{array}}PD\left(i^{\prime },j+l,k^{\prime }\right),\;\mathrm{and} \end{array}$$

(2) $$\begin{array}{cc} \; & BPD\left(i,j,k;l\right)=\max_{\begin{array}{c} i-l\leq i^{\prime }\leq i+l\\ k-l\leq k^{\prime }\leq k+l \end{array}}PD\left(i^{\prime },j-l,k^{\prime }\right), \end{array}$$

where PD(i,j,k) is initialized with the value of its normalized density

(3) PD(i,j,k)=D(i,j,k).

#### 2.2.2. Accumulation and Reverse Pyramid Influence Zone

The accumulation proceeds iteratively through intermediate voxels $\left(i^{\prime },j^{\prime },k^{\prime }\right)$, whose PD is updated from immediately adjacent voxels in the previous *Y*-slice (i.e., $j^{\prime }-1$ for the forward direction or $j^{\prime }+1$ for the backward direction) according to

(4) $$\begin{array}{cc} \; & PD\left(i^{\prime },j^{\prime },k^{\prime }\right)\;=\;D\left(i^{\prime },j^{\prime },k^{\prime }\right)\;+\\ \max_{\begin{array}{c} m,n\in \{-1,0,1\}\\ (\mathrm{if}\;\mathrm{contributing}) \end{array}} & PD(i^{\prime }+m,j^{\prime }\mp 1,k^{\prime }+n), \end{array}$$

where ∓ denotes the minus for Equation (1) and plus for Equation (2) and only neighbors m,n∈{−1,0,1} within the above search pyramid are contributing (see Figure 4 of [19] for an illustration). This scheme allows only voxels in a reverse pyramid to influence the blue target ($i^{\prime },j+l,k^{\prime }$). The “if-contributing” condition in Equation (4) forces an intersection of the reverse influence pyramid with the above search pyramid, yielding the red accumulation zone in Figure 4A.

### 2.3. Combining Forward and Backward Path Densities for Filament Pattern Enhancement

In the second preprocessing stage, the FPD(i,j,k;l) and BPD(i,j,k;l) values are combined to form a single map, where (i,j,k) acts as the center point and FPD(i,j,k;l) and BPD(i,j,k;l) are sampled from the two opposite directions. It is expected that if voxel (i,j,k) is located on a filament segment, it will have high values for both FPD(i,j,k;l) and BPD(i,j,k;l).

The two directional PDs can be combined using a blending function. In our original approach [19], we used a simple product for this purpose (first in the following list), but in the present paper, we also explored three additional alternative blending functions. Consequently, one of the following four equations was used in the present paper:

(5) $$\begin{array}{cc} CPD(i,j,k;l) & =FPD(i,j,k;l)*BPD(i,j,k;l),\mathrm{or} \end{array}$$

(6) $$\begin{array}{cc} CPD(i,j,k;l) & =FPD(i,j,k;l)+BPD(i,j,k;l),\mathrm{or} \end{array}$$

(7) $$\begin{array}{cc} CPD(i,j,k;l) & =\sqrt{FPD(i,j,k;l)*BPD(i,j,k;l)},\mathrm{or} \end{array}$$

(8) $$\begin{array}{cc} CPD(i,j,k;l) & =\min\left(FPD(i,j,k;l),BPD(i,j,k;l)\right). \end{array}$$

The CPD values are also normalized to a range from 0 to 1 for an easier way of classifying them in subsequent stages of the workflow (Figure 1). Therefore, no normalization constants appear on the right side of the equations.

The original multiplication (Equation (5)) provides a heuristic score to ensure a logical conjunction (*and* gate); only if both FPD and BPD are large will CPD be large as well. In this type of blending, the product of the two densities in the filtered map CPD no longer corresponds to the density of the biological specimen (e.g., the larger dynamic range might amplify inhomogeneous density variations).

The addition (Equation (6)) is identical to the arithmetic mean (we ignore any normalization constants). It appears to be a more natural way to combine accumulated (summed) densities BPD and FPD. Moreover, the filtered map CPD has the advantage of being proportional to the physical density of the specimen. Addition is similar to a logical disjunction (*or* gate), so filament voxels (with simultaneously high FPD and BPD) are rewarded less than by multiplication.

Because Equation (5) is essentially the square of the geometric mean (ignoring normalization), we can take its square root (Equation (7)). The geometric mean (Equation (7)), much like the arithmetic mean (Equation (6)), is a physical density (not density squared), but like Equation (5), it acts as a logical conjunction because filament voxels with simultaneously high FPD and BPD are rewarded more than surrounding noise (albeit at a compressed dynamic range because of the use of the square root).

Finally, we also tested the minimum function (Equation (8)). Like the geometric mean (Equation (7)), it is a density and acts as a logical conjunction (because filament voxels with simultaneously high FPD and BPD are rewarded more). However, the dynamic range of the minimum function is not immediately obvious and requires further testing on actual density maps.

### 2.4. Candidate Seed Point Selection

It is often convenient to initiate automatic tracing from a given set of seed points. For example, Sazzed et al. [10] required the user to provide a seed point for each filament in a highly regular (hexagonally packed) actin bundle. Rusu et al. [9] used a genetic algorithm to find seeds for bidirectional tracing of isolated, irregular filaments. In particular, the manual placement of seeds is a tedious process and is only feasible when a small number of filaments are present. For hundreds of actin filaments forming loosely organized bundles with variable spacing among them (Figure 2), a manual seed point selection is not practical (and it is also subjective and not reproducible). The automated genetic algorithm search uses cylindrical templates that require the filaments to be well separated [9] and, therefore, is not applicable to our case of intermediate packing density.

The newly developed CSP generation stage of our workflow (Figure 1) involves a spatial subdecomposition of the map into cubes of a user-defined size. For each cube, the voxel with the highest density value is considered a CSP. Here, we used cubes of 5 × 5 × 5 voxels. (The CSP cube length was identical to the path length, *l*, hence providing a natural length scale for coarse-grained seed placement.)

All the local high-density voxels in the spatial decomposition are initially considered CSPs; however, it is not yet known whether any CFS generated from them constitute true filaments because the final traces are determined later (Figure 1). A direct determination of true seed points and corresponding true filament traces is computationally out of reach because of the low signal-to-noise ratio and missing-wedge artifacts present in tomograms [1]. Therefore, as described in the next section, the algorithm first generates a large number of CFSs from all the CSPs. Later, the CFSs pass through several rounds of screening to determine the true filaments (Figure 1).

### 2.5. Tracing of Candidate Filament Segments

From each CSP, we can trace a path of length *l* (the same length as above) in the dominant forward direction (+Y axis in this work) to generate a set of short CFSs. Longer filaments will be fused from the short CFSs, if indicated, at a later stage. A CFS is represented by $\left(\left(i_{s},j_{s},k_{s}\right)\to \left(i_{e},j_{e},k_{e}\right)\right)$, where $\left(i_{s},j_{s},k_{s}\right)$ is the start voxel (i.e., the CSP), $\left(i_{e},j_{e},k_{e}\right)$ is the end voxel (i.e., the voxel with the maximum FPD after tracing a path of length *l* from $\left(i_{s},j_{s},k_{s}\right)$ along the forward pyramid), and $l=j_{e}-j_{s}$.

The CFS tracing can use either one of the forward processing algorithms shown in Figure 4. We have already explained the DP method (Figure 4A) above (Equations (1), (3) and (4)) because it is used in the preprocessing of the map. In the current paper, the relatively narrow width of the accumulation zone, as shown in Figure 4A, inspired us to test an alternative straight-line density accumulation (Figure 4B) that is simpler to implement. In this alternative approach, instead of considering the density of the neighbor voxels (Equations (3) and (4)) for creating the CFS ($\left(i_{s},j_{s},k_{s}\right)\to \left(i_{e},j_{e},k_{e}\right)$) of length *l*, we can consider straight lines, where lines are drawn from each of the seed points $\left(i_{s},j_{s},k_{s}\right)$ to the target points $\left(i_{e},j_{s}+l,k_{e}\right)$. For a specific seed, the target point $\left(i_{e},j_{e},k_{e}\right)$ is the point on the base of the search pyramid, $i_{s}-l\leq i_{e}\leq i_{s}+l$ and $k_{s}-l\leq k_{e}+l\leq k_{s}+l$, that exhibits the maximum FPD (Equation (1)) among all the base points. The intermediate voxels of each straight line are determined by interpolation of the X(Y) and Z(Y) indices, here using first-order Lagrange interpolating polynomials in *Y*, rounded to the nearest integer.

In both approaches, we compute the $FPD(i_{s},j_{s},k_{s};l)$, and we also identify the corresponding CFS end voxels $\left(i_{e},j_{e},k_{e}\right)$. For each CFS, we finally obtain its NPD (normalized FPD) score, with a range between 0 and 1. (The FPD values range from 0 to (l+1) because the densities D(i,j,k) were normalized between 0 and 1, so we divide by (l+1) to obtain the NPD; this revised formula supersedes the earlier calculation [19]).

### 2.6. Grouping and Selection of Candidate Filament Segments

Based on their NPD scores, the generated CFSs were grouped into 10 bins of a width of 0.1. Therefore, the bin numbers reflect the first floating point digit of the NPD values (e.g., bin 10 contains CFSs with NPD scores ranging from 0.9 to 1.0).

We observed earlier that CFSs of very high-numbered bins usually represent segments of true filaments. As we gradually move toward the lower-level bins, many false filament segments appear. For example, in our simulated map, we can see that the CFSs belonging to bins 6–9 are primarily true filament segments, whereas the CFSs of bins 5 and lower exhibit false filament segments that are no longer localized in the expected filament region of the map (see Figure 6 in [19]).

To identify the bin that starts introducing false CFSs, which we refer to as the threshold bin, we take advantage of the fact that false CFSs in the threshold bin spread to the full volume (Figure 6 in [19]) because they are mainly picking up noise. By iterating from high to low numbered bins, we automatically detect at which bin value the CFSs are no longer localized and spread to the full volume. We decompose the tomogram into 100 × 100 × 100 voxel cubes, which is an intermediate level of detail between the fine CSP grid and global map size. If we find that at least 15% of the cubes contain less than 10 CFS midpoints, we deem that the CFSs do not yet occupy the entire volume, and we proceed to test the next lower bin. In this way, the approach selects the top bins that represent mostly true CFSs. Note that the threshold bin number is not a static value; it may vary based on the density distribution of the map. For example, in our simulated tomograms, we have observed various threshold bin indices when different levels of noise are added.

Subsequently, in another refinement step, the CFSs of the selected bins are further screened based on backward tracing. Backward tracing helps determine whether a CFS truly represents a filament segment because it is expected that the traces of a filament should be similar in both directions. Specifically, we select the CFS endpoint from forward tracing and retrace it backward using BFD. False CFSs are then excluded based on their dissimilar forward and backward trace orientations (angle tolerance: 30${}^{\circ }$). Because the algorithm is sufficiently fast to generate CFSs, this retracing step does not introduce any significant computational overhead.

### 2.7. Fusion of Filaments

The final stage of filament formation employs multiple strategies to fuse surviving CFSs into longer individual filaments:

*Connecting CFS by collinearity:* This step considers the collinearity to connect adjacent filaments that exhibit the same orientation and represent fragments of the same filament. A pair of CFS that are collinear or nearly collinear (0 to 6${}^{\circ }$ angle) and are very close (or connected) along the primary axis of the filament (distance between 0 to 10 voxels) are assumed to represent the same filament and are consequently merged to generate a single, longer CFS.

*Removal of isolated CFS:* An additional screening step automatically excludes spatially distant CFSs that are arbitrarily generated because of the presence of noise in the tomogram. A small number of false CFSs can exhibit moderate to high NPD scores. If these CFSs are indeed caused by erratic, local noise and not by nearby filaments (that are populated by other CFSs), these spurious CFSs can be detected and excluded by considering the mutual separation of CFSs in a local region (Figure 7 in [19]). To determine whether a CFS is false (isolated), its center is first computed, and then, the number of other CFSs present within a sphere of a radius of 20 voxels are determined. If the number of neighboring CFSs is less than three, the CFS is considered false and excluded accordingly.

*Extending and fusing the CFS:* Because of the noise present in the cryo-ET map, a filament may not exhibit a homogeneous density distribution along its length. Therefore, it is possible that the fragments of the filament (i.e., CFSs) fall into multiple bins; some may even fall below the threshold density and are excluded in the above screening. To bridge between such weaker segments of the same filament, we extend the surviving CFSs as follows: Each CFS of length *l* is gradually extended in the forward direction by repeatedly adding a new segment of length *l* but only if this new segment has an NPD score of at least that of the threshold bin. This process continues until the NPD value of the newly generated segment falls below that of the threshold bin or until a maximum length of 5l is reached (this empirical limit caps the number of overlapping traces, which will need to be reduced below, but the exact multiplier of *l* has little effect on the final results).

The current set of CFSs is then fused using a directional traversing algorithm. Specifically, we labeled all voxels on the CFSs that survived the previous steps as filament voxels (FVs). Starting from one FV as a seed, we iteratively traverse in the main filament direction (+*Y* in this work) by considering that the possible range of movement along the *X*-axis and *Z*-axis is half of the *Y*-axis movement. For example, using relative grid indices for an FV (0,0,0) and connection range of value 2, the algorithm first checks whether voxel (0,1,0) is an FV; if not, it checks whether any of the voxels (0,2,0), (0,2,1), (0,2,−1), (1,2,1), (1,2,−1), (−1,2,−1), (1,2,0), or (−1,2,0) are FVs. If a new FV is found, it is selected as a second voxel of the FS, and the search continues until no FV is found, which marks the end point of that FS. Then, we initiate traversing again from another FV, which does not belong to any of the existing FSs yet, and follow the same procedure. This process continues until all FVs are assigned to their final FSs.

*Excluding redundant filament segments:* This final refinement step excludes short FSs that overlap with a longer FS along the dominant axis of filaments. By discarding the spurious FS, this step can also help distinguish true filaments from noise artifacts. FSs that have more than 90% voxels in common with a longer FS are automatically discarded (Figure 8 in [19]).

## 3. Results

Below, we provide molecular graphics visualizations of the resulting maps and filament tracings. This is followed by a quantitative statistical performance analysis. The validation shows how downstream tracing differs if different path density accumulations or denoising preprocessing are used. We conclude the section with information about the program run times.

### 3.1. Visualization

**Figure 5:**
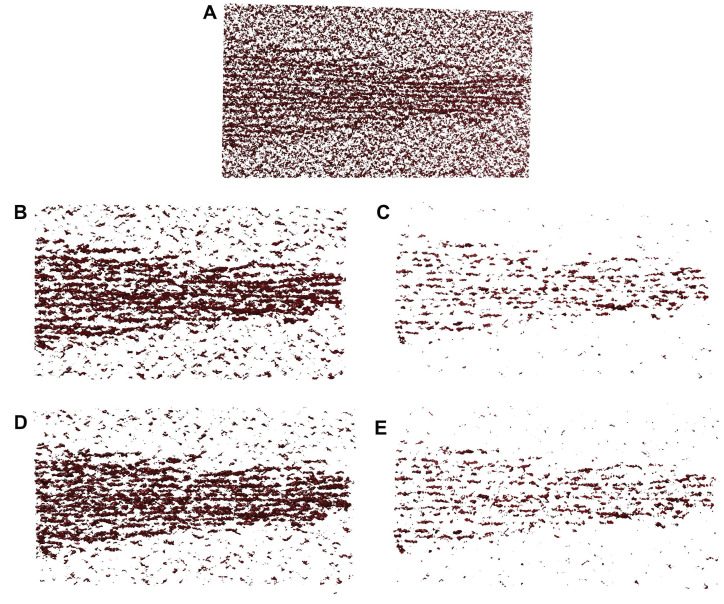
Comparison of the unfiltered and various CPD-filtered maps using l=5 voxels. The same 10-voxel-wide slab of Figure 3C) is shown, except that it is rendered at the mean + standard deviation isolevel to emphasize the noise. (**A**) Original unfiltered map with a noise level of 0.8 (as in Figure 3C). (**B**) The map filtered by multiplication of FPD and BPD (Equation (5)). (**C**) The map filtered by addition/arithmetic mean (Equation (6)). (**D**) The map filtered by the geometric mean (Equation (7)). (**E**) The map filtered by the minimum (Equation (8)).

Figure 5 shows a comparison of the original map and maps after DP-enhancement of the filament pattern. In the original density map (Figure 5A), the distinction between the density levels of filament and noise is not always obvious; thus, computationally separating them may not be possible. In the CPD maps (Figure 5B–E), the filaments are enhanced and more distinguishable from the noise. The visual inspection at a relatively high noise level of 0.8 shows that all four methods can be useful as denoising filters.

**Figure 6:**
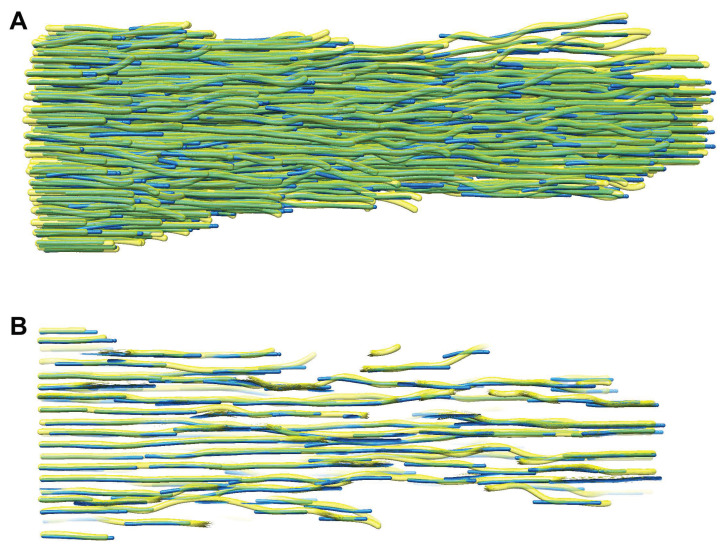
Automatically detected FSs (solid blue; this work) superimposed by the ground truth manually traced filaments (transparent yellow, [18]). (**A**) The green areas in this full-view rendering indicate good agreement because of the subtractive color mixing. (**B**) A slab consisting of 10 *Z*-slices, which is taken from the center portion of (**A**), provides a detailed view of the individual filaments. The FSs (blue) have been obtained from the 0.60 noise level simulated map (Figure 3B), which is closest to the automatically matched noise (see the Methods section), here by using DP-based enhancement with multiplication (Equation (5)), DP-based CFS tracing, and l=5.

The final FS results of one typical case are compared with the ground truth manual tracing in Figure 6. The persistence length of pure actin filaments is on the order of 10 μm, which is more than three orders of magnitude longer than the length l=5 voxels, or 4.7 nm. Nevertheless, some of our traces in Figure 6 clearly follow curved paths that are picked up by the tracing algorithm on short scales that justify the use of l=5. This is not surprising because the curved filaments on this scale have been detected by manual tracing (Figure 2), and there may be a biological interpretation—for example, because of the cross-linking of filaments and the decoration of filamentous actin with other proteins.

As of the different normalized CPD distributions, it is hard to predict from Figure 5 which denoising strategy will enhance the filament density most relative to the noise level. Therefore, we have performed a quantitative analysis of the tracing performance of these four cases in the following section. Quantitative validation is also important for ruling out any errors introduced by the positional and directional granularity of our approach. (Given the short CFS length l=5 voxels and restriction of CFS end points to voxel positions on the 3D grid, our approach is limited to about 9${}^{\circ }$ directional and 1 voxel = 0.947 nm positional granularity).

### 3.2. Statistical Performance Evaluation

To assess the performance of the proposed tracing framework, an *F*1 score–based statistical measure is employed. The criteria for the *F*1 score calculation are similar to those used by [24], except predicted FVs are dilated by one voxel to balance recall and precision values (see below). The ground truth FVs determined by the manual annotation [18]) are compared with those of the automatically traced filaments. True positive (*TP*), false-positive (*FP*), and false-negative (*FN*) voxels are defined as follows:

*True Positive*: A predicted FV is considered a *TP* prediction if a true FV exists within 3 voxels in any direction.

*False Positive*: A predicted FV is classified as an *FP* voxel if no true FV exists within 3 voxels in any direction.

*False Negative*: A FV in the ground truth map is considered an *FN* if no predicted FV is found within 3 voxels in any direction.

Based on the computed TP, FP, and FN values, the recall (*R*), precision (*P*), and F1 (F1) scores are calculated using the following equations:

(9) $$R=\frac{TP}{TP+FN}$$

(10) $$P=\frac{TP}{TP+FP}$$

(11) $$F1=\frac{2\times P\times R}{P+R}$$

Table 1 shows the precision, recall, and *F*1 scores of the proposed framework when applied to the simulated tomograms of various levels of noise and using different processing methods. As a performance reference, the exclusive DP-based approach (Table 1, left) provides a very high *F*1 score of 0.97 for a simulated map of a noise level of 0.4. The very high recall score (0.99) suggests that the framework identifies almost all the filaments present in the simulated tomogram at this noise level. As the noise level increases, it negatively affects the performance, even though the obtained *F*1 scores can still be considered very good. Even at a noise level of 1.0, the DP-based approach shows an *F*1 score of 0.86, suggesting that it is capable of detecting filaments in the worst-case map (see the Methods section), albeit with some minor inaccuracies. With the current settings, the precision score is slightly lower than the recall score in the pure DP approach (Table 1, left) because of the remaining FPs, as can be expected given the noisy nature of the tomogram. Nevertheless, the observed F1 scores from 0.86–0.95 (for noise levels 0.6–1.0) are quite high for a density-based structure prediction. For comparison, in a recent state-of-the-art deep learning prediction of secondary structure features in cryo-electron microscopy maps, we achieved *F*1 scores of 0.72 for alpha helices and 0.65 for beta sheets [25].

Table 1 also provides a comparison of the proposed DP-based framework to two alternatives workflows, which are explained in the Methods section, one with an alternative filament tracing based on straight-line density accumulation (Figure 4B) and one without the filamentous pattern enhancement (denoising).

Substituting the line-based tracing in the workflow (Table 1, center) significantly lowers the precision and *F*1 scores compared with the pure DP approach, mainly because of a significant increase in the number of FP filaments. The results suggest that (at least with the current settings) any efficiency gain by the simpler approach comes at too high of a performance cost. Because of the apparent overinterpretation of the noise, we did not implement the line-based tracing in the more expensive preprocessing stage, where noise suppression is crucial.

For a better-quality map (0.4 and 0.6 noise level), the denoising (Table 1, left) provides only a modest benefit (compared with Table 1, right) and may not be necessary. However, at higher noise levels the detection breaks down (Table 1, right). Unless denoising is used, no filaments are detected for noise levels ≥0.8 (at least with the same parameter settings as in the other cases).

Table 2 shows the performance of the proposed DP-based framework with the alternative addition/arithmetic mean (Equation (6)), geometric mean (Equation (7)), and minimum (Equation (8)) blending functions. The results correspond to those of multiplication (Equation (5)) shown in Table 1 in the left column. As can be seen in Table 2 (center, right), the performance of both geometric mean and minimum degrades at higher noise levels. This is because the compressed dynamic range of the density distributions impedes the binning and associated screening of CFSs, such that many FN filaments remain undetected, which then lowers the recall. Only the arithmetic mean blending manages to achieve similar *F*1 scores. However, the high value at noise level 1.0 is likely an outlier because the trend at lower noise levels is irregular, as exemplified by low recall values at noise level 0.8 (which exhibits binning problems because of the lower dynamic range). At least with the current binning approach, multiplication remains the most consistently well-performing blending function across all noise levels.

### 3.3. Algorithm Run Times

The tracing part of our framework is computationally highly efficient, but the preprocessing step (which is a requirement for noisy maps, as indicated in our *F*1 score analysis) takes more time. On an Apple MacBook Pro with a 2.6 GHz Intel Core i7 processor, it takes around 3 min to trace filaments in a simulated tomogram with a size of 283 × 664 × 269 voxels. In contrast, the denoising of all voxels by the preprocessing stage is slower, taking about 5 h. The tracing stage of the filaments is much more efficient than the denoising stage because the coarse-grained selection of the CSPs is based on the highest density voxels in the spatial subdivision. For l=5, the CSP generation selects one voxel out of 5×5×5=125 voxels, whereas the preprocessing acts on all the voxels in the map. Moreover, in the preprocessing, each voxel is traced twice: once in the forward direction and once in the backward direction, whereas in the tracing stage, only the forward direction is required.

## 4. Discussion and Conclusions

In the current work, we have optimized a fully automatic and fast framework for tracing filaments in an semiregular actin bundle. Our DP-based approach is a spatial domain technique that operates directly on the voxels of the 3D tomogram. The use of simulated maps based on a known model has allowed us to validate the algorithm performance quantitatively. The main result of the statistical analysis is that the earlier approach described in a previous workshop paper [19] is robust, and it would take some considerable effort to match or surpass its performance.

The neighborhood-based density accumulation scheme (Figure 4A) enables robustness in tracing because we do not observe clean filament densities that could be picked up by a thin line accumulation (Figure 4B). The pyramidal search window ensures that the PDs are large when filaments pass through the tip of the pyramid while providing some robustness against noise because individual voxel densities are replaced with PDs. The tracing assumes that filaments are oriented in a mean direction and bundled, even though individual filaments may deviate up to 45${}^{\circ }$ from the main direction because of the search pyramid. The main advantage of the current approach is its applicability to dense filament bundles while still being able to follow individual curved filaments.

Among the various blending functions, the multiplication of forward and backward path densities provides for an efficient filter lifting the filament density above the noise level. Our results (Table 1) show that such a denoising is crucial for the detection of filaments in lower quality maps. There are alternative denoising filters already in use in tomography [26,27,28], but generally, the earlier filters have made no assumptions about the shape of the biological structures. For example, the earlier work of Starosolski et al. [26] also considered a path density–based filtering, but numerically expensive random walks were required to sample the density map isotropically. In contrast, our bidirectional filter is designed for filaments that are mainly oriented in the mean direction of the bundle, and we could take advantage of this known direction to develop a more efficient approach.

The tracing stage of our framework is several orders of magnitude faster than our earlier methods and only takes minutes (facilitated by the spatial coarse graining of the CSPs). However, the denoising of all tomogram voxels is a current bottleneck and still takes several hours on a standard computer. Note that in both the forward and backward directions, density is accumulated from the origin (initialized in Equation (3)). Therefore, DP is performed only locally for each voxel, and the full density map needs to be processed exhaustively, which is expensive. We will explore further speed up in the future.

Prospective experimental applications [1,10,18] of our *Spaghetti Tracer* framework call for a modeling of filament gaps, as described in Figure 2 of [18], and for a more detailed analysis of filament curvature. Another future application of our framework would be the tracing of irregular filaments that do not exhibit a mean direction [9]. The future DP algorithm we envisioned [29] would be able to identify high-density filament segments in any arbitrary orientation by combining the above DP search pyramids in ±X, ±Y, and ±Z directions into an omnidirectional search cube. To enable future work on more complete cellular tomograms, we have already developed a spatial subdecomposition [1] scheme for handling large 3D maps in memory, and we have taken the first steps to generalize the tracing to the detection of other cellular components besides actin bundles [1,18].

## Figures and Tables

**Table 1:** A performance comparison of the proposed DP-based framework with the line-based approach for tracing actin filaments without density enhancement preprocessing at various levels of noise. UND = undefined because the *FP* and *TP* values are both zero.

Noise	DP-Tracing			Line-Tracing			DP-Tracing		
	w/DP-Enhancement			w/DP-Enhancement			w/o Enhancement		
	Pre.	Rec.	*F*1	Pre.	Rec.	*F*1	Pre.	Rec.	*F*1
0.4	0.945	0.994	0.969	0.591	0.988	0.740	0.963	0.909	0.935
0.6	0.923	0.978	0.950	0.603	0.962	0.742	0.952	0.878	0.913
0.8	0.848	0.965	0.903	0.568	0.940	0.709	UND	0	UND
1.0	0.828	0.898	0.861	0.575	0.813	0.674	UND	0	UND

**Table 2:** Performance achieved with both DP enhancement and DP tracing using addition/arithmetic mean (Equation (6)), geometric mean (Equation (7)), and minimum (Equation (8)) blending functions.

Noise	Addition			Square-Root			Minimum		
	Pre.	Rec.	*F*1	Pre.	Rec.	*F*1	Pre.	Rec.	*F*1
0.4	0.945	0.994	0.969	0.950	0.982	0.966	0.946	0.99	0.968
0.6	0.948	0.806	0.806	0.967	0.250	0.397	0.95	0.35	0.514
0.8	0.926	0.686	0.788	0.936	0.176	0.296	0.938	0.444	0.602
1.0	0.939	0.879	0.907	0.874	0.138	0.239	0.904	0.139	0.241

## Data Availability

The prerelease source of *Spaghetti Tracer* can be freely downloaded at https://situs.biomachina.org/fflavors.html.
